# AmI and Deployment Considerations in AAL Services Provision for Elderly Independent Living: The MonAMI Project

**DOI:** 10.3390/s130708950

**Published:** 2013-07-12

**Authors:** Jorge L. Falcó, Esteban Vaquerizo, Luis Lain, Jose Ignacio Artigas, Alejandro Ibarz

**Affiliations:** Grupo de Investigación Tecnodiscap, I3A, Universidad de Zaragoza, María de Luna 1, Zaragoza 50018, Spain; E-Mails: evaqueri@unizar.es (E.V.); llain@unizar.es (L.L.); jiartigas@unizar.es (J.I.A.); aibarz@unizar.es (A.I.)

**Keywords:** AAL, ambient intelligence, assistive technology, e-inclusion, elderly, mainstream technology, autonomy, services provision

## Abstract

The MonAMI project aims to investigate the feasibility of the deployment of open platforms for Ambient Assisted Living (AAL) services provision based on Ambient Intelligence (AmI) and to test user acceptance and the usability of the services. Services were designed to provide support in the areas of environmental control, security, well-being and leisure. These services were installed and evaluated in a Spanish geriatric residence. The participants included elderly persons with disabilities, nursing home care givers and informal carers. The concept of the open platform proved to be satisfactory for the provision of the services in a context aware framework. Furthermore, the usability of the technology was viewed positively and the overall results indicate that this system has the potential to prolong independent living at home for elderly people with disabilities. Deployment was proven successful and awareness of open-platform AAL service delivery was raised in local communities throughout Europe.

## 1. Introduction

The fact that life expectancy is increasing and the resulting ongoing growth of the older adult population, have led to new models of aging which seek to empower people to have fulfilling lives in the dwelling of their choice, being this a nursing home or private home. Independence is a critical issue not only for older adults, but also for people with disabilities who may wish to remain at home and increase their quality of life.

The use of assistive technology is a successful strategy to help promotion of independence and health maintenance [1,2]. Despite these positive outcomes, access to assistive technology is not widespread yet. Availability of assistive technology is achieved by ensuring that the needed infrastructure, personnel, products and materials are available. Appropriate assistive technology should meet users' needs and environmental conditions providing sustained services at the most economical and affordable price.

Assistive technology has a powerful support in AmI technologies, due to the interoperability, use and sharing of contextual data, ubiquitous computing and natural interfaces provided, as well as directly embedding and integrating devices in the environment. AmI technologies are increasingly finding use in assistive and medical applications in locations such as hospitals and homes of elderly people, as described in [3,4].

Both quality of life and economic impact have been widely demonstrated in the field of health care [5]. In the assistive field, AmI implementation lacks large studies that could address the assessment of improvement in the quality of life and economic efficiency together with feasibility versus local restrictions throughout Europe [6].

The literature shows different aspects in AmI for AAL that are relevant in current AmI status, namely interoperability framework and contextual information sharing, feasibility of large scale deployment, interfaces and alert handling as well as user modeling for autonomy assessment. These issues will be dealt with within the following sections and taken later as reference in this communication to describe design decisions and results of the MonAMI project.

### 1.1. Interoperability Framework and Contextual Information

AmI architecture regarding application in assistive technologies can be simplified into different moduli: acquisition and processing contextual information by sensor networks; specific health sensors with different levels of intrusiveness; middleware frameworks that boost interoperability and offer contextual information available to several applications; user interfaces, with local and remote operation and notification functionality; alarms and risk event detection, handling and notification; intra-home communication networks with external servers, other homes/service centers or mobile devices; actuators inside the home; and leisure devices or terminals, which may coincide with the formerly mentioned interfaces.

Several efforts have been made to link the contextual information of a sensor network [7–9]. One option that has been used successfully in health monitoring and AAL is OSGi, an open framework that supports interoperability, as in Uranus [8]. OSGi offers a semantic abstraction and fast heterogeneous devices integration via bundles that was also proved suitable for the application in the MonAMI project.

Environmental information, together with user information and parameters of usage of the system, facilitates comprehensive connections of devices to users through the middleware architecture. Practical interoperability is achieved. Moreover, integration of contextual information can enable the system to recognize unusual or dangerous situations and to anticipate alerts for health problems or special user needs in a technological living environment, such as a house or a public space as in [9].

Some other works on this topic include a “cluster cultures” approach which aims to support heterogeneous technologies (including legacy ones) from Twente University [10] which provides for interoperability in a hierarchical structure approach and from Waseda University [11], with a Virtual Service Gateway through a several protocol translation. Based on translation, we also find the Building Information Exchange Group [12] using XML and Web Services; the “Domotic House Gateway” [13] also translates sensor events to virtual semantic events that can be standardized and shared in the contextual information frame. Amigo [14] and universAAl [15] are other implementations proving similar interoperable structures.

The MonAMI project has focused specially on the feasibility aspect of AAL. On the one hand, it has tried to integrate mainstream devices, achieving a modular AAL system built with interoperable devices. This provides for s lower price and wider access to technology. On the other hand, it has paid special attention to the service value chain and its integration in the community and its infrastructures: services have been developed attending to a simplified Tahi value chain, so that several modules can be easily combined for several services and with other mainstream modules. It has also attended to local restrictions as far as culture, different needs, legal framework and technological infrastructures available; it has also looked into the micro-ecology of the local services, having studied and identified the different agents in the community that would maintain it in service, such as manufacturers, telephony providers, caregivers, maintenance personnel, *etc*. It also has focused on the feasibility of large-scale deployment.

Interoperability frameworks in the future will advance towards a more comprehensive semantic approach [16,17], which will enhance user friendliness, context awareness and sharing of information among heterogeneous systems. Ontologies (such as OSGi) and Semantic Web will most likely improve semantic tools to exploit knowledge representation of the environment [17], aiming to a better understanding of the user needs and preferred use of the system. These will also aim to provide with dynamic adjustment of system rules to environmental conditions so as to achieve specific goals. This has also been studied and anticipated in guidance systems [18], and assistive AmI environments [19]. The results obtained from development of these frameworks will possibly be applied to the medical field, specifically to anticipate health problems [20].

### 1.2. Feasibility of Easy and Large Scale Deployment

Attention to the large scale implementation is considered a critical feature: “Integrating AmI developments into full-scale systems is not an easy task” [21]. Implementation of Funblocks [21], addresses those issues looking for equilibrium between completeness and practicality. They select a minimalist modular framework for the development of AmI systems, based on the function module abstraction used in the IEC 61499 standard for distributed control systems. As in MonAMI with OSGi, Funblocks proposes a framework for the development of AmI systems through the integration of modules loosely joined by means of an event-driven middleware and a module and sensor/actuator catalog, which is very similar to the MonAMI approach.

Some authors go further in this concept [22], stating that “conventional software and mainstream systems should be integrated with the pervasive services and the smart space”, as well as considering and enhancing the advantages related to simplicity, useless innovation, compatibility, network limitations, and level of abstraction. For this reason, in MonAMI this has been stated as a main starting goal.

During installation, introducing topographical information of devices and sensor location makes it a burden regarding simplicity and cost of installation, limiting the concept of an self-installable kit. Some experiences have been reported in this aspect [16,23].

Attention to the service provision chain is considered crucial when considering large-scale deployment feasibility. For this latter aspect, the Tahi value chain gives a detailed decomposition of a service that allows for study of better options for service delivery and aggregation [24].

### 1.3. Interfaces and Alerts Handling

AmI has a large potential for detecting abnormal situations, risks events and emergencies, based on its high information integration. Some works have got deep into this aspect, highlighting the importance of such events, not only from the user needs perspective, but also from its critical handling and interface information displaying [25,26]. Design in [25] attends specifically the interfaces for interaction between carers and the AAL system, showing the importance of giving support to carers on checking the situations inferred by the system (alerts, subject's location and activities, *etc*.) in a friendly and intuitive manner [27], including the availability of explanations for such situations, both textual and visual. A nursing home is a singular context for caring, in which coordination of caregivers and traffic of critical information is basic for the well-functioning and quality of the caring [26]. Necessity regarding problems to be detected and solved is further studied in [28].

### 1.4. User Model for Autonomy Assessment

Selection of data for user modeling is a challenge in any experimental knowledge building action, in order to be able to relate results with works already performed. Also, to leave data obtained in a format that can be usable by future related works. AAL AmI initiatives need to relate obtained results information to user's profiles, to share and re-use results and enlarge significance of population by comparing and integrating results from other experiences, allowing also for trans-cultural comparison. Characterizing user profile is not a direct task: we find in the literature several heterogeneous classifications from different sources that make it difficult to extract large population conclusions [29]. Syntactic and semantic heterogeneity of user models need to be addressed in order to enable user modeling interoperability. A dynamic user profile structure is proposed, based in Simple Knowledge Organization for the Web (SKOS) to provide knowledge representation for ubiquitous user model. Of course this is not the only field where this classification has to be made; similar approaches can be found in health data [20].

### 1.5. MonAMI Objectives

The European Commission has set up several activities under the 6^th^ Framework Programme (FP), which have been continued under the 7^th^ FP to initiate a Europe-wide dialogue among all parties working for an accessible and inclusive information society. MonAMI: Mainstreaming on Ambient Intelligence [6] was a five years long project, funded under the 6^th^ Framework Programme by the European Commission, with 14 European partners and a budget of 13 M€. MonAMI [30,31] focused on:

- Capitalizing on AmI technologies to ensure that the services can be used without behavioral change.
- Building on top of mainstream devices and services such as TV-based Internet, nomadic devices, *etc*.
- Doing initial experimentation in Feasibility and Usability Centers and subsequent large-scale validation in Validation Centers in five countries.
- Addressing economic viability and long term sustainability of such services in large communities in different Member States.

MonAMI selected ranges of services in the areas of comfort applications, communication/information, health, safety and security. It built, tested and deployed these services and demonstrated that they can be economically brought into the future mainstream ambient intelligence technologies. MonAMI focused on services, platforms and usability: The technology platform was derived from mainstream technology. Usability requirements were identified, an evaluation methodology was selected and usability analyses were carried out.

The objective of the MonAMI project was to demonstrate that accessible, useful services for elderly and disabled persons living at home can be delivered using mainstream systems and platforms, making context aware systems applicable to AAL field. This was done in close cooperation with users and institutional stakeholders and by involving key mainstream actors throughout the whole process.

The project had to address all practical issues that arose when designing and implementing AmI AAL services in living environments by the hand of the local institutions, elders, families and caregivers implied, including infrastructure support, service providers and manufacturers.

This report also describes the work carried out at the Living Scaled Field Trial (LSFT) site in Zaragoza, Spain, to test the MonAMI services and technologies in a living environment. It is intended to contribute to the body of knowledge concerning the testing and usefulness of ambient assisted living (AAL) services and technologies for persons with disabilities, elderly persons, their family and friends who care for them and the nursing home caregivers. Main findings are a positive proof of concept of open architectures for elderly service provision, with a high impact both on: (1) the exploitation and marketability, (2) the service update and new functions development and (3) a potential support for improvement of quality of life for the final user and caregiver. It has shown potential as alternative and complementary solutions for dependency and its associated social cost and impact in quality of life.

### 1.6. Structure of the Paper

From the literature, four main key issues have been identified in AmI for AAL, which have been used to structure the introduction, together with some general AmI considerations and an introduction to the project which actions we are describing.

In the following Experimental section, those key issues are revisited, highlighting the design decisions taken in the three main types of actions that the project has carried out: development, user needs and evaluation management and deployment. This Experimental section is completed with a description of the project actions and methodologies used for each one of the actions: specifications of architecture and services, technology development with services description and services description tools developed, and validation and assessment through deployment: functionality and usability tests in six countries, local reliability testing and in-site usability, living scaled field trial to assess deployment and impact in real living conditions and the way data has been gathered automatically as contextual information.

The Results and Discussion section shows the main results considered in each of four key points highlighted. It also shows the results obtained addressing multidisciplinary collaborative work with specific guidelines and functional description tools and users feedback. The local creation of an AAL community integrating most actors in the ecology of service provisioning to elders is also described as a positive result. Finally, the Conclusions section summarizes the main findings and appreciation by local stakeholders. It also addresses future actions foreseen in different areas.

## 2. Experimental Section

### 2.1. Design Options in AmI for AAL

The MonAMI project was driven by three main threads, which concentrated in needed aspects to achieve its goals. On one hand, the project focused on users' management and attention, specifications gathering and evaluation design of usability and impact on users' life and wellbeing. On the other hand, there was technology development coordination. Those two threads link in the services, specified and tested by users and developed and modified by technology.

One more thread was deployment, this is, implementation and integration attending local circumstances and actors (ecologies) of service provision, having real demonstrators as a proof of concept. Thus, actors such as local governments, companies and professional workers that could make use of the system and benefit from services were taken into account, as well as other service provision companies with which the project services collaborated or competed, building significant and sustainable value chain structures. This one thread also pays attention to large deployment feasibility, and real mainstreaming devices and software integration. Services sustainability is dependent on such features, as backed up by other author's work in the introduction, and in our own experience when deploying the living scaled demonstrators. Legal framework, other deployed technologies like tele-alarms or home-IP communications, culture and weather conditions and social network support through family or neighbors are ultimately as important as good technology design to be able to keep a full system working that is able to provide health and autonomy support to elder people in general and dependant persons in particular.

#### 2.1.1. Interoperability Framework and Contextual Information (Technology Thread)

It is shown in the introduction that interoperability in AAL needs a framework that integrates mainstream devices and software and provides for context information sharing both for final applications and for intelligent inference motors. Those drivers may obtain behavior patterns and notify when deviations may mean alterations in health or well-being.

MonAMI established as one major goal to choose and/or develop an open framework that provided for modularity and interoperability of mainstream devices and software, so services could be built on top of whatever sensors and actuators the location would have available. Integration of areas in which service provision is given to particular homes was also a major concern and main goal, including health, social support, environmental interaction support and leisure. By taking advantage of context information, MonAMI decided to maintain an open door to any innovation through connection with a specific bundle and translation into the defined semantics. The OSGi open framework was selected for compatibility and interoperability, and UCH was chosen for the generic interface.

**Figure 1.**
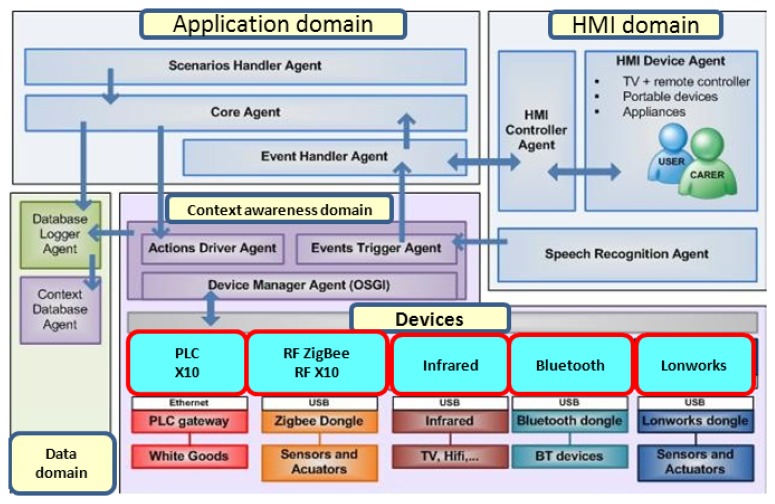
Architecture. Description of technical implementation based on the OSGi architecture. The picture shows different technologies integrated into the system: PLC, Bluetooth, Zigbee^®^, Lonworks or Infrared.

Mainstream software included communication (such as telephony functions or Skype™) and web browsers. Mainstream hardware included sensors (wired and based on Zigbee^®^, Bluetooth and X10) from different manufacturers, as shown in Figure 1. MonAMI proposed and used a framework for the development of AmI systems based in OSGi through the integration of modules loosely joined by means of an event-driven middleware and a module and sensor/actuator catalog.

MonAMI built a context domain which can be fed by different heterogeneous inputs and used by different applications using its semantic approach. During the project, information came from the user interaction, from processed images that located the user indoors and had a fall-detection implementation, door and window open/close sensors, movement throughout the house and environmental sensors collecting light, temperature and humidity information. Moreover, with parallel projects, the same system was fed with the internal state variables of the white goods (“Easy Line +”, European STREP project) and identification of food and medicines by integrated RFID readers, and health measurements as heart rate, blood pressure, glucose level in blood, among others (“Centro de Salud Virtual”, a Spanish national project).

Inference from information of different sensors, re-scaled in time referenced with get up time and eating times of each day proved later to offer the possibility to build more understandable context information regarding use of the system and behavior patterns of the user.

The architecture supports interoperability and context awareness by its design, including mainstreaming devices connectivity. On top we can find a “logic domain” or an “application domain” and a “Human Machine Interface (HMI) domain”. In the application domain any application has access to context information, provided in a standard way by the context domain. The context domain, in turn, takes information from any plugged device through the device manager agent, which provides an abstraction level of contextual information making it available in a standard OSGi format. Connectivity is shown in the figure with examples of Power Line Communications (PLC), RF-X10, ZigBee^®^, Bluetooth, Infrared, lonworks, with which the platform reached sensors. In MonAMI, complemented with a Health Care project, the platform had actuators for lights, doors, windows, shutters and switches connected through ZigBee^®^ and PLC, presence or movement, temperature, light, doors and windows sensors connected through ZigBee^®^ and RF-X10, Medical devices and leisure devices connected through Bluetooth, TV remote connected through infrared, standard domotics connected through Lonworks. Interconnectivity and modularity was proven, which was one of the goals at platform level.

This platform fulfills AAL requirements about interoperability among heterogeneous devices for mainstreaming integration, which means at functional level:

- modularity (we can add or take out any device as needed to adapt to final user special needs).
- context awareness (any information any device is reporting is added in a standard and “understandable” way and made available to all applications.
- price reduction: as the platform can integrate any mainstream device, a modest system can be built up from medium-cost readily available technology, and also a full and complete one with high quality devices (as happens normally in connected medical devices).
- accessibility: it also improves the burden of finding and buying a specific system, for mainstreaming devices are normally easier to find and substitute.
- adaptability: any new service can use information and actuation for any device in the system, so new services can be built up sometimes at a very low added cost (for example, when needed sensors are already deployed, like in movement sensors installed for security, and used later to monitor abnormal movement patterns in the house). Configuration is also easier for we can rely on parallel information (e.g., light sensors and shutter opening in the morning).

All this interoperability can be made transparent to the user. Carer and cared are both interacting over the system on the HMI domain, which in the MonAMI project was offering an adapted PDA with voice recognition, touch screen to monitor and control the environment, to set preferences, to raise and receive alerts and to ask for specific information. It also had the standard UCH/URC protocol included, in a cooperation with the i2home project which facilitated the inclusion of their HMI protocol.

One more domain is the data domain, from which applications can retrieve state variables and perform usage and habits studies with stored data.

#### 2.1.2. Feasibility of Easy and Large Scale Deployment (Deployment and Technology Threads)

**Figure 2.**
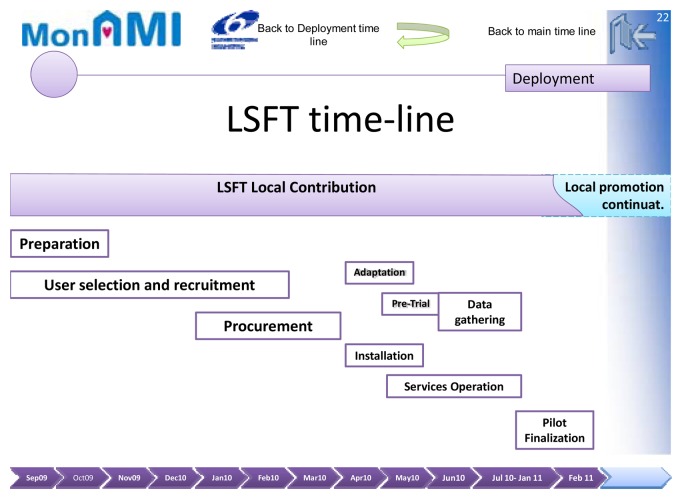
Demonstrator system implementation logistics in time.

MonAMI attended deployment and feasibility of services continuation by placing the value chain and the local ecology of the system inside the main focus (Figure 2). For this reason, real demonstrators were envisaged from the very beginning, doing a task that later the EC Office would place in a different framework, AAL and then CIP programs, recognizing its large importance.

This action, carried out in three European countries, Slovakia, Sweden and Spain, gave as a major result the feasibility of deployment in three different legal and ethical frameworks, three different technologically evolved environments and three different cultural and institutional realities. As far as technology is concerned, Sweden had at that time domotics as a social service and tele-alarms based on IP telephony. In Spain telephone-based tele-alarms were the major social support service for dependents, with a large effort in regulation of the legal framework for well-being of dependency situations. Also, IP communications showed a lack of reliability and quality of service which forced us to use redundancy to raise events and alerts. In Slovakia tele-alarms had recently been implemented, and IP systems were present in the large cities, which was where the demonstrators were located. In all three cases the final implementation had modifications due to technological issues such as IP available infrastructure, ethical or legal issues related with the culture and country and users preferences. Varying the services given and originating specific services deployed on a local level in the living conditions was also needed. Some examples are IP medicine intake reminder (Sweden), surveillance at the entry door of the dwelling (Sweden); weather forecast information system and remote assistance to close shutters and protect from storms (Slovakia); Time Orientation devices to adapt to nursing home rhythms and activities (Spain); mainstream communication software and leisure games in the system (Spain).

Real mainstreaming devices and software were integrated in the system, as standard smoke and gas alarm sensors, medicine intake reminders, communication software and leisure games and activities. Also for interfaces and internal communications, mainstreaming devices were integrated regularly or as a proof of concept: smart phones with regular communications, redundant sms and internet for alerts and picture taking (only proof of concept). In parallel projects, white goods and medical diagnosis devices were integrated to the same platform.

#### 2.1.3. Interfaces and Alerts Handling (Users and Technology Threads)

**Figure 3.**
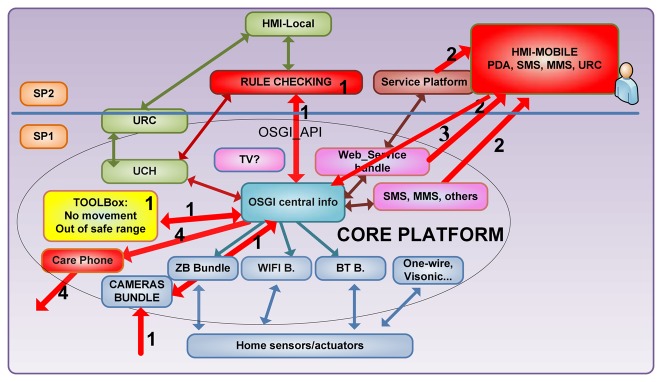
Service communications mapped on technology blocks allowed for inter-team understanding and Quality of Service and responsibility of faults assessment. Tele-monitoring and monitoring service.

MonAMI supervised security of the user by different sensing strategies and raised alerts to be checked by a human actor when singular events were detected. MonAMI addressed the study of quality of service of communications and responsibility of each system building block. MonAMI used service decomposition based in the Tahi model [24], with the main purpose to build business and exploitation schemes that could re-use existing services structures and so made more feasible the cost of service provision. In parallel, responsibility of functioning of each building block was made clear. Even if MonAMI chose not to address at that stage any critical alarm, it had the potential to supervise the status of a person and raise alerts, as happened during the field trial in Slovakia, where a son was informed of abnormal situation in his father's house on the mobile and was thankful to had been able to reach in time to address the situation. The scheme shown in Figure 3 was designed as an internal project communication tool among technological teams and user-management teams, and was proven useful also to address studies of quality of service and eventual responsibility of faults if a service information chain should fail.

Checking and agreeing on the information paths not only by technical staff, but also by expert users was found necessary. The process of seeking an understanding among expert teams of different profiles made clear the differences in language and mental patterns of each one, so graphical information of the different moduli and information flows among them was found necessary. The flexible service design based on the moduli already existing in the system also made it necessary to provide, in a graphical and functional way, the sets of devices that every service would use or gather information from. Moreover, when reaching decisions of implementation (or not) of each service, the maturity of each building block again showed the need of moduli functional description for non-technical professionals.

Figure 3 shows the example of modular description of a monitoring service for a person with dementia. This tool shows the different functions performed in time (numbers mean order in sequence) and which building block is performing the function or passing the information, which gives an overview of functionality and of modules needed, alternative paths, reliability in relation with reliability of each step.

In the example, the user scenario is a home of a couple in which the carer has gone shopping leaving her cared at home. Previous to departure, the carer has connected the surveillance service, so a camera is sending a stream of images which are received through a CAMERA bundle and ZigBee bundle.

Number 1 shows the entrance of information in image form, which goes to TOOLBox through the OSGi kernel configuration, because the surveillance service is activated. Toolbox detects a pattern situation and raises an event which is heard by rule checking (logic domain, in Figure 1), an application in any case. In this case, a potential fall, unusual activity or abnormal absence of activity is detected, so in STEP2 the carer is informed through an alert. Figure 3 shows different paths the alert raising can take, through an external service platform (like a tele-alarm central), directly through a web_service bundle or directly through mainstreaming communications like sms or mms.

In step 3, the carer asks for more detailed information, such as a picture of the room where an event has been reported. This information also flows through a server, interacting with a web page or come via sms. Finally, the carer may take the action to make a phone call directly through the care phone, which communicates with OSGi platform in such a way that generates contextual information and acts over an external communication media.

#### 2.1.4. User Model and Autonomy Assessment (Users Thread)

MonAMI had to find a balance between different goals: seeking for significance of results in the impact in quality of life and autonomy on the user's lives on the one hand and testing interoperability framework significance and flexibility with different and heterogeneous technologies and services on the other hand.

For users' validation, there were still two options that required balancing: to try as many services possible with the maximum amount of people's profiles to obtain an overview of what can be efficient, usable and acceptable, or to try just a few with restricted user profiles to seek statistical significance in the impact on their quality of life.

Finally, there was one tendency to reduce the number of services to a few, and try and have less user profiles to obtain larger groups seeking for statistical significance. The other tendency integrated increased services and wide user profiling: increasing the number of services test platform flexibility in service creation and provision increasing the heterogeneous devices and integrated software and try and have different user profiles to obtain a first evidence of usefulness for each group.

Reality saved the project from the effort to make such decision, as user recruitment finally didn't get the resources needed to accomplish the effort of seeking a reduced set of profiled users. Also the set of services available for installation was naturally reduced by the budget and maturity of development when the installation deadline was becoming firm.

No adequate profiling was found in literature that the project could use for preparing the project results to be shared in AAL literature, so some *ad-hoc* global personal and sociological profiling was performed, including items such as gender, rural or urban location of the dwelling, nursing home or particular home, if the person lived alone, with a spouse, and a set of characteristics about the capacities of the people.

The project also had to address local interests, such as compliance of profiling with local regulations. Locally in Spain we were asked to provide results to the local government under the format of the Spanish dependency law, so we opened a profiling line that was consistent with the project's one and complemented and translated data into that format. To gain generality we made trials using the International Classification of Functioning, Disability and Health (ICF) from the World Health Organization, having very good results in translation to other formats and good local acceptance that will be further described in the Results section, being considered a good option for AAL user profile sharing.

### 2.2. Project Actions

#### 2.2.1. Specifications

MonAMI used several methodologies to raise and check service specifications in search of efficiency to achieve better autonomy and well-being. Creative work sessions were held with technical and user teams. Consulting was done with experts from user associations, with manufacturers and companies providing services in the field, with local people responsible for service provision such as local governments and city-halls. Derivation studies were made to obtain new services from existing ones, by combination or addition of new features, contents or communication channels among actors.

Analysis and accessibility assessment was performed, prior to the selection of services to be implemented in the living trials. In order to put information in place, also “User Fit” [32] templates were used. User Fit was found complex for the practicality and requirements of our project, so a simplified tool was designed, as shown in Tables 1 and 2.

User fit had different templates to be fulfilled to check requirements at the user level and at the activity level. This was performed in a scenario, with which contextual information was completed. UA1 (User Analysis) and UA2 of user fit took the capacities of the user and how those affected functionally the system requisites. This is summarized in UA3 as desired product characteristics for each studied user profile. All three templates are summarized in our first table, which condenses user profile attributes and extracts directly functional implications and desired product/service features. In case conflicting requirements appear, a column is set to identify them and another to set priorities to be able to decide among potential conflicting requirements.

In Table 2 AA (Activity Analysis) templates of user fit tool are condensed. AA1 sets the list of scenarios and identifies the activities to perform in each. AA2 takes each activity identified and details functional implications and desired product characteristics, while AA3 again identifies possible conflicts and gives priority information about them. Table 2 shows a template per each scenario and directly activities identification, functional implications, desired product characteristics, conflicts and priorities.

Internally in the project, user-management teams and responsible people for the local deployment presented requisites to technology developers, which are extracted in the following lines:

- Interoperability of applications related to devices: Make transparent for the application layer how a temperature sensor (e.g., provides the temperature so that they can benefit from one or another without changes).
- Interoperability of application related to residential gateway: This goal states that services developed should be able to run on “the two telecom platforms in the project” without the need for modifications, so that there is no need to make compatible software versions on which they run.
- Common OSGI interface: the data structure of the devices, services and applications are interchangeable as far as technology is concerned when the same function is performed (e.g., domotics), implementing a pre-agreed ontology that has been developed by subproject of technological development and subproject of deployment management together.

Another important criterion for service promotion was its inclusiveness. MonAMI project stated three major points to assess it:

- Accessibility
- Compliance with necessary functionalities for the user: user requirement study
- Innovation features

#### 2.2.2. Technology Development and Services Description

The MonAMI technology base for delivering the services is the MonAMI platform developed from mainstream, open-source components with a touch screen computer as the central element. Other parts are a Universal Control Hub as the user interfaces server, wireless sensor networks and a remote service management function. The result is a platform that is flexible enough to deliver a wide range of different services and facilitate future development and addition of services in a cost-effective manner (see Figure 1).

The services developed by the MonAMI project have been grouped into five packages: AMiSURE for safety and security, AMiCASA for home control, AMiVUE for home status information, AMiPAL for time management and information giving and AMiPLAY for games.

The selected services were first tested in six Feasibility and Usability Centers with user tests in lab-like conditions. The Centers have different profiles and address different user groups. For example, the Slovak Center focused on analyzing and enhancing the integration of inclusion services based on mainstream technologies in new EU Member States.

Once the services and applications were found to be feasible, usable and appropriate to user needs, a living-scale field trial was carried out at sites in Slovakia, Spain and Sweden. Many users tried the services in their homes and the impact and consequences have been analyzed. The economic viability and long term sustainability of the services has been addressed in order to facilitate real mainstream implementation.

#### 2.2.3. Service Description Tools

**Figure 4.**
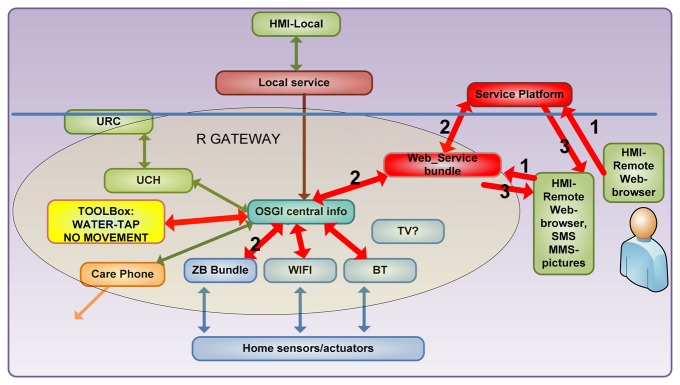
Service communications mapped on technology blocks allowed for inter-team understanding and Quality of Service and responsibility of faults assessment. Information on demand from a relative to monitor a dependant person and check an alert received.

As mentioned before, MonAMI developed a modular-functional tool to describe services and allow fluent communication among internal working teams. Figure 4 shows another example of information flow. It was proven useful for understanding and adapting information flow by the user and the technological team.

The figure is complemented with tables that finally map each functional component with the devices that support its functioning, showed in Tables 3 and 4, based in decomposition tables: Services decomposition into software and hardware components, which show which devices and pieces of software where needed for each service at each layer of the architecture, including application and middleware. Maturity progress of each component development or/and procurement process was put in the table, so we could readjust reliability and establish redundancy.

In Figure 4 another surveillance service is described. The carer takes action 1, asking through a web browser or a service platform some information on his/her house. OSGi detects which devices are to give basic information to elaborate the piece of information asked for. In step 3, information is prepared and given back to the carer.

This way, functionality was mapped onto diagrams in Figures 3 and 4 and then further mapped onto devices and functional units of software code with Tables as 3 and 4, having a common description tool used by all teams in the decision taking of dropping, keeping, starting or enhancing a service, specifically when deciding about money-service efficiency or time managing restrictions.

#### 2.2.4. Deployment and Validation Assessment

##### Functionality and Usability testing

In the MonAMI project, assessment was divided into two specific actions. In the first one, involving usability, functionality and feasibility testing, the goal was to check previewed usefulness and usability of the services by a set of users with different profiles and from different countries in Europe. Six sites were set to test functionality and usability of the services in six different countries throughout Europe.

Results showed a lack of usability of interface solutions and large modifications that needed to be made showing large differences in preferences stated by users with physical, sensorial or cognitive disabilities. Result feedback was incorporated into the design and development process to adapt the necessary modifications.

In Spain, two main different setups were deployed: one in a residence of people with physical disabilities (from Aragon Physical Disability org), and a second one in a training flat for independent living, which belongs to ATADES: a large organization taking care of cognitive disability throughout all ages in life. We also performed some testing in special education schools.

A clear conclusion was the need of a protocol to address the planned actions, performed by third persons that were not part of the team of researchers, and in which some faults we detected could also be included, such as talking with the relatives, waiting for arrival of ethical permissions, contacting local institutions to start building local AAL culture, promoting diffusion, *etc*. More on this can be found in [33].

##### Reliability Testing and “Usability-In-Site”

We had carried out various reliability and functionality test to ensure that the system is ready to be installed in the home environment. The tests were carried out in our laboratories, by qualified staff. All incidences were recorded. Having studied the test reports we identified the issues to be addressed and modifications that were necessary before continuing to the next set of tests. The actions taken depended on the relevance of the issue, and the possibility of modification within the project scope (e.g., limited time and resources). The tests were carried out on the integrated system, as it would be installed in the home environment.

After this internal verification of the system, the device was put under stress/fatigue testing, that is, temporary operation above the normal demands to detect possible failures due to stress/fatigue and increased communications rate.

When the result of this first stress/fatigue testing was found satisfactory in the laboratory conditions, the devices were installed in two pilot flats located in Valdespartera (Zaragoza), where they were run in a real life environment together with other devices. In this environment they were also exposed to new factors/conditions such as changes in voltage, failures in net connections, *etc*. In this controlled environment (yet closer to a real life environment), trials were carried out with some carers and potential users. The trials provided us with usability data on the system and its services.

When the reliability, functionality and usability tests were passed, we installed the services in real home environments in a non-occupied room at the Residential Home Romareda, where functionality tests were carried out by users, including caregivers, who used the system and its services for a given time. Finally, services were installed in occupied rooms and they were used in regular living conditions.

Reliability tests were described and reported by the partners providing technical support for the field trial. Functional tests were also performed and several incidences reported to and addressed by the technical support and the technical staff. These tests led to significant improvements concerning usability (especially the user interface) but at the cost of delays that impacted on management, installation and user training.

The devices had to be adapted to the real conditions at the Residential Home Romareda where the users who were participating in the field trial would still ask for minor changes and configuration patterns. Installation also had to take into account that this site was the home of people and so the installation was already studied to shorten times and adjust to their schedules. There is a limited and defined physical space (placement of equipment, electricity, connections…) and time available for the trial. Also, the trial must not cause disturbances in the nursing home. Therefore, we carried out three types of testing in the trial:

- Testing with the nursing home caregivers in the users' unit.
- Testing with care staff from the entire home.
- Testing with residents, carers (informal) and users.

##### Living Scaled Field Trial testing

**Figure 5.**
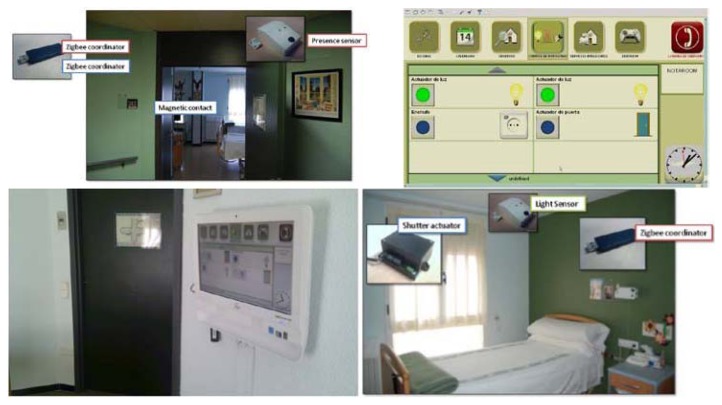
LSFT installation: The pictures show some of the sensors integrated into the LSFT as well as the Human Machine Interface in the rooms.

The Living Scaled Field Trial (LSFT) in Zaragoza was carried out in a nursing home owned and managed by the local government. This site was chosen to gain an insight in the deployment of AAL services in this scenario as well as to provide information concerning usability and acceptance of ICT support services for independent living. Technology installed and interface used are shown in Figure 5.

There is an identified “independent living gap” that makes a difference in quality of life and social expenses in the transition of people from their own homes to nursing homes. MonAMI set pilots in both sides of the gap: in the homes of elderly persons living independently in their own apartments/homes in Sweden and Slovakia and in nursing homes in Spain for elderly persons. As such, the LSFT in Spain intended to provide insight on the acceptance, usability of AAL services by people at the least autonomous side of the gap.

The selection of participants was user-centered. The participants in the trial were 15 elderly persons with disabilities (users) living in the Romareda residential home (nursing home) in Zaragoza, their carers (2) and caregivers (7). Four main factors were considered in the selection of services to be tested in the LSFT:

- Results of the feasibility and usability testing, final reliability testing as well as consultation exercise with external experts concerning their evaluation of how services could affect the different evaluation dimensions.
- Adjustment to project goals, as framed by the evaluation design, to facilitate collection of information in evaluation dimensions that is comparable over the three LSFT sites (e.g., feasibility of larger numbers, using scenarios with carers or not, nursing homes or private dwelling, *etc*.).
- Budget: Ability to comply with the budget available for the project.
- Local needs: Taking into account individual LSFT sites and stakeholders' resources and expertise.

The MonAMI LSFT was the culmination of much of the work of the MonAMI project and provided the opportunity to field-test services developed within the project at three European sites. It was designed as one pilot project implemented in varying infrastructures at different locations in different configurations around the core set of MonAMI services. This aimed to provide information for a coherent analysis with the additional potential for the comparison across geographically and culturally separate contexts.

##### Gathering Evaluation Data

The MonAMI architecture is prepared to use a log mechanism to store and report log messages generated by the MonAMI platform and each of the services. The log entries are used to detect issues for debugging (reported to the service providers itself) and to analyze the usage of the services. The OSGi system and the respective service providers implemented a common log mechanism.

Each of the log entries was stored locally on the user gateway. They could be consulted in real-time using the web console. For off-line analysis, a MonAMI service took the log entries and sent them periodically by e-mail. The period and the e-mail address are configurable and were configured by the installer.

Each LSFT site had a data repository for the log of service use. This log was developed such that each time a MonAMI service was employed in each home, data on the time and exact service were transmitted and filed back at the site. The log only recorded the User ID, so that the participant's identity was unknown to the staff member collecting the log data.

The purpose of the log was to record the usage of a particular service. Any issues can subsequently be addressed either through re-configuration or removal of the service (at the users' request). The log was also designed to investigate any sustained periods of under-use or user-abandonment of the service. The log is a quantitative measure, which can be used to support the users' self-assessed, anecdotal acceptability (or non-acceptability) of the services.

## 3. Results and Discussion

Services described were implemented and deployed, having run for four months during the project scoping, they run later for four months at the Romareda Nursing Home of the Aragon government.

### 3.1. Interoperable Framework and Contextual Information

Feasibility of the open interoperable framework was proven satisfactory, having provision of services, which used devices from the market and from different manufacturers, working in a middleware with semantic abstraction in which contextual information was shared. Also *ad-hoc* devices and mainstream software were integrated.

Specially, new services development was proven fast and flexible, by integrating resources available in the middleware. Also configuration of the developed services was quick, although the interface to do this didn't achieve a final fully usable version during the project.

Integration of services of health, physical autonomy support, cognitive autonomy support, surveillance, leisure, tele-monitoring, tele-operation and communications was achieved, showing to the local AAL communities the concept of a single home platform for service provision at an affordable cost (being this cost referred to the basic set, increasing modularly with the different services and moduli incorporated).

A semantic approach in which applications used personal and environmental contextual information were deployed in real life for service provision in AAL, sharing information of light sensors, temperature, movement and presence, gas or smoke detection, flood, cameras, detectors of door or window open, to name but a few. On top of them, applications to monitor life habits such as raising shutters in the morning, ventilating the room, control of light level, surveillance, home control and others were built and tested successfully.

Single sets were proven in six countries, and living complete sets were deployed in three countries finding the support of three regional governments to implement living pilots, proving also trans-culture acceptance through Europe. The set also proved that AAL middleware based on open platforms can support medical and security fields, complemented with other projects that developed this other technologies on top of the same system.

### 3.2. Feasibility of Easy and Large Scale Deployment

Large deployment feasibility was proven, at the regular cost of electrical installations in the market and with an investment in procurement of equipment, which was considered very minor by the local government representatives compared to the benefit provided. Still difficulties with geo-location and identification of each sensor impeded installations from reaching the level of a self-installable kit that was aimed to promote an open market and make it more economically affordable. Anyhow, continuation of services and finalization of services did produce several incidences and considerations that are worth reporting.

A major goal of the project was to study and promote continuation of services beyond the time period the project was financed, specific attention was paid to it, and several key parameters were identified, paying attention to the ecology to which the services were to be incorporated, and the sustainability of the procedures of deployment in a large scale. This ecology (elements which live in a sustainable system and their interrelation) was seen from different perspectives by different experts in the consortium, fact that help ed identification of several dimensions in it: stakeholders related to use, stakeholders related to financing, services and technological structures, all of them actors in the value chain.

- Stakeholders related to use: management of the nursing home; nursing home staff (caregivers); final user and their informal carers (normally spouses); families. The management of the nursing home saw the project as interesting and with potential to improve the life quality of people and caregivers. The caregivers showed varying degree of motivation towards the use of the system. Re-structuring of personnel in all social services in Aragon was performed around those dates, so some may have seen technological services entry related with it, which may have largely influenced in the motivation to promote the use. In MonAMI we also found the importance of the families, both in positive and negative acceptance of the services: some discouraged the use of the systems when they felt they have not been explained enough and they had not had an opportunity to ask about them; also some were highly motivating actors when they found services useful and increasing the security and/or comfort of their older relatives. Especially teleconferences and e-mailing relatives was a large motivating factor.
- Stakeholders related to financing: local government bodies have large influence to promote financing of AAL services through regulation actions and direct financing. Private companies as banks have some specific financing capabilities through social actions in Spain.
- Stakeholders related with service provisioning: Security companies and tele-alarm companies are willing to include AAL services in their portfolios. An emerging service provisioning sector of private companies is also taking protagonism in this field in Spain, linked with care and food delivery.
- Technological structures: Communication infrastructures are a key point of services delivery when talking about perceived security and surveillance, even though many other autonomy and support services can be delivered in site. In rural areas where communications externally to the village is limited due to cost, AAL services are finding good interaction with voluntary and neighbor common-assistance networks. Here, trans-generational support is maintained and enhanced and groups of elders take care of each other. In any case, benefits of social interaction are enhanced.
- Other existing services: Every service put in place may interact, conflict, or enhance other existing services.

In the MonAMI project it was found important to prepare previously the interaction of the service-system with each of the elements of the ecology described. The Tahi value chain and service provision chain was used and found valuable. It was found too complex for practical and dynamic implementation, so a simplified version of it has been used, integrating several layers in a few functional blocks and reducing the used structure to four elements.

Regarding installation and maintenance investment, our project sought sustainability, although some circumstances made it difficult to try and get a simulation of real maintenance: the first installation step was done in about two hours per room, having to adapt to the dwelling's timetables. The second installation step was done in another two hours per room, although the configuration was not proven enough and we learnt as we configured services, taking much longer than we would expect in a regular mainstreaming installation. Also accompanying was necessary due to the fact that reference caregivers were substituted, so in those specific situations our researchers became somehow the reference regarding the technological services deployed. One full time researcher was accompanying the users through the experiment and visiting to make tests and repeat trainings or change configurations when necessary.

Training was given after a baseline questionnaire was passed. It was first given to caregivers in an empty room, which had the same installations as the others. Then, researchers accompanied and observed how caregivers trained elders. In many situations this plan was not feasible due to the substitutions of the caregivers, so the same research personnel trained elders as well. Their improvements in skills and capacity of use and reconducting the system was observed and recorded. Quantification of used hours is not a significant measure in this case, for we couldn't simulate the planned implementation as mainstream services provision.

Training was organized with a manual that guided the navigation through the information, selection of services and their operation. As services were incorporated progressively as a smooth training strategy, training was repeated iteratively, allowing researchers to check the permanence of skills and training.

Regarding un-installation, our project had to face a non-natural situation: services were put in place for some elders, and then taken away. As far as ethics we couldn't sort out the un-ethical withdrawal of services that the elders found useful. Even when we had worked together with the department of social affairs of local government and they were convinced and willing to promote and invest those services and platforms, elections changed the people responsible and the actions were cut.

Another important aspect considered and still found later is the risk of installation of technologies under development that are not available in the market after the project. Installation was made in two phases: the first one accomplished the installation of the system after the project ended, foreseen some technology would be retired due to incapacity of maintenance when those were not commercial mainstreaming products. The second phase accomplished the installation of sensors and devices as needed for the trial, and this phase had to be reversible to the first one at the end of the project. Installation and de-installation were subcontracted to external companies that would maintain the repairs or maintenance guarantee beyond the time period covered by the financing of the project.

A previous study was done of the elements that were repairable or substituted by the nursing home maintenance service. At the end of the project, after a fault in prevision that led the installation being inoperative for a weekend, development elements were disconnected and mainstream pieces were put in place, giving the manuals to maintenance service to allow them to procure and substitute any element. Finally, as the service was discontinued, the main features were just turned off.

### 3.3. Interfaces and Alert Handling

MonAMI service definition found major user needs relating to real security and perceived security at home and elder dwelling, both for the final user and the caregiver or relative.

Regarding alerts, pop-up windows were designed and direct messages to mobile devices, carried by the staff of the nursing home, were sent. They had to go to the main floor control place where the central computer was to access to contextual information to check the event that was signaled.

Again, external factors largely influenced the use and validation, for a large re-structuring of staff inside local government was performed and only one caregiver was kept from the original trained and motivated team the project had worked with, so we had to start all over and they were incorporated in the middle of the trial.

The interface was redesigned twice to make it more clear and attractive, and a contest was performed among designers to get a better one. Icons were finally easy to locate and functions that were missed by users in the usability and functionality tests were included.

### 3.4. Users Model for Autonomy Assessment

As mentioned in the experimental description section, in Spain we had some local requirements that made us try different forms of profiling users to place results of the trials in different formats, specifically in dependency law format. We also worked with different user associations and found they used different tests to profile capacities of their associates, being Barthel among the most frequently used.

We got the support from our local government to make a parallel study, with some extra financing and some actions based in games and time orientation inside the nursing home. We tested ICF classification and made translation to the Spanish dependency law and project internal formats:

- It was proven useful and gradually complete; we could choose the level of detail wanted for each item. Comparison between studies with different level of detail in each items were possible by reducing the levels in a homogeneous way.
- Translation into regular tests used by local user associations was feasible, although the large amount of items in the taxonomy made necessary an introductory course that was held in our university. Also translation from medical records was tested. As ICF also considers health (it incorporated the ICE10 international classification of health alterations used in the medical field) this exercise proved very useful to maintain consistency with nursing home records, and again, their own internal information structure.
- Accessibility assessment was performed in a very balanced way, even compared with accessibility regulations, where cognitive accessibility is not addressed with the same weight as sensorial, for example.

A course was held to promote ICF awareness. Different user groups representing elders, user groups representing people with disabilities (physical, cognitive, sensorial and social), people from institutions (local government and city hall), dependency-certified evaluators, private professionals and university researchers received and participated with practical exercises. The majority of the learners conveyed the usefulness of having a common tool that allowed for this comparison. Posterior modifications of Spanish dependency law were also inspirited by ICF, so compliance was further increased.

### 3.5. Interdisciplinary Working Tools and Guidelines

Understanding among deployment responsible researchers, technology and development, and user needs and evaluation has been a challenge in this large project. An action that made it easier, as it facilitated communication and common understanding, came from the need to have a common language and a common and written idea of the services the project aimed for.

Services were the meeting common point of both teams, for they offered the functionality the users appreciated and they needed the technology the developers understood, so the service description tool, described in the Experimental section of this communication, proved to be a crucial communication tool for technological and non-technological people involved in the project. The insight of the complexity and information paths that each service had underneath, together with information related to cost, maturity of development and procurement status allowed to take decisions jointly by the three teams in critical timing difficulty periods of the project.

Building up or designing new services at the block level was a task that could also be performed by a non-technological researcher, which supported the input for user's perspective in the design of new services, together with the understanding of the increased cost and complexity/simplicity of the new add-on.

### 3.6. User's Feedback

The MonAMi trial ran for four months and had 15 users in Zaragoza's Living Scaled Field Trial. Baseline questionnaires were passed before any training started with the users, focusing in Quality of Life and capacities profile of the user, and including some economical parameters. Then at the end of the project those baseline questionnaires were repeated to check differences and opinion surveys were passed about the utility, accessibility, efficiency, attractiveness and will of buy. Other teams of the project have published some descriptions of the evaluation and feedback results that can be found in [34].

Some users felt the system provided some support, appreciating autonomy features such as raising and lowering of shutters and having little impact on security, as was foreseen. Others reported they valued the system offered, and had learnt to write e-mails and establish videoconferences with their cared ones. Most have appreciated the leisure offer, asking for more type of games and establishing small social networks centered around the rooms where they could play, reporting also that they had felt more awake and were in a better mood. Their conversations had also changed into more positive and socializing.

Among caregivers, different opinions were also reported. Their participation had mainly been reception of notification of incidences and observing the use of the system. Re-structure of staff in the middle of the trial influenced the acceptance of the system by the users, because elders depended affectively on their caregivers and many had been gently pulled by this affect to participate in the trials, so frustration came in the middle and our user team had the need to cope with it together with the specific trial.

### 3.7. Final Overview

- *Compatibility of the platform with other existing and necessary platforms for service delivery (*i.e., *health and social services)*: This is clearly an advantage that the MonAMI open platform offers. Moreover, in the frame of Spanish national projects we are linking medical diagnostic devices at home to this same platform.
- *Demonstrate the flexibility of the MonAMI system to add and update services efficiently (both convenient and with significant cost reduction)*: local institutional stakeholders we have worked with are convinced of these advantages of the MonAMI system.

During the MonAMI project we contacted the majority of user associations in our region, and had continual collaboration with the largest ones. With this action, we have promoted the concept of an open platform for delivery of AAL services with focus on interoperability and mainstreaming, and culture of cooperation in finding new solutions, integrating capacities and perspectives of implied stakeholders. During MonAMI project, a small local AAL community was created, which acted as a reference group, supervising and suggesting changes in services, validation methods, scenarios and timing.

## 4. Conclusions/Outlook

In this work a deployment and a proof of concept of the use of open platform for services provision has been demonstrated. The local government in Aragon has found it positive, being specially interested in the modularity, openness and flexibility to integrate health and social services. The research team also considers that the proof of concept of usefulness and deployment of the technological architecture and service provisioning system has been a success and has large potential to lower costs of equipment by introducing mainstreaming technologies. Awareness of the importance of the value chain, open platforms, interoperability, modularity and mainstreaming has been raised by a large extent. AAL community has been promoted and common assessment tools initiated.

The local government in Aragon has found the proof of concept of a single and interoperable home platform for service provision positive, stating the interest and willingness to migrate the experience to particular flats and counseled flats. They have also found of great interest the assessment via ICF, promoting a parallel project to validate its applicability and the extension of its potential.

Integrating different technologies from different manufacturers via semantic middleware has proven very positive. Facility to change or add services has been a large advantage of current technological system. Further development is needed to reach an off-the-shelf solution with more mainstreaming options by extending the number of bundles for interoperability that the system has. It is also a matter of further quantity.

The project has succeeded in raising awareness of the importance of integrating services and having awareness to the value chain, open platforms, interoperability, and modularity among the local stakeholders (government, organizations, industry) in Zaragoza. Having gained the interest and support of the stakeholders, new local projects came after this one to further prove usefulness of specific services and complete the deployment/installation tools, and also to extend benefits to other groups (e.g., to demonstrate the extent independent living can be supported by ICT, to demonstrate the economic impact ICT services can have on the cost for support).

Several local stakeholders such as local public/private institutions and user organizations who showed interest in the MonAMI services in various sectors were moved into a temporary AAL community. Regular meetings were held with these stakeholders to check the possibility for integration of the services in schools, nursing homes and homes for persons with disabilities and training/counseling institutions.

The combination of increased awareness of the importance of horizontal actions with a clear vertical market and a local AAL community are major achievements that the MonAMI project has reached at the local level. This may stimulate AAL services in the community and support more horizontal and standardization actions in the future. We consider the positive LSFT trial as a proof of concept of open architectures for service provision in a stakeholder ecosystem which implies the elements of the service provision chain and the primary stakeholders (carers and beneficiaries) and the quaternary stakeholders (political institutions). The creation of a local AAL community triggered by the development and testing of the services and the deployment of the LSFT, together with rising of awareness of the role technology can play, have been important successes.

Regarding user models, the use of a world-wide standard such as ICF has been successfully used in AAL field, and has been proven useful for accessibility assessment, user capacities profiling, and autonomy assessment, as far as functions in which the person may show improvement, among other areas.

Tools for collaborative understanding and working among multi-professional teams have been developed and used, proving to be useful and efficient. Future actions with ICF potential are planned.

Future actions regarding technology are inclusion of different mainstreaming wireless and wired protocols, and building up new services on top of the technological structure and contextual information gathered. Regarding applications, new scenarios are to be considered, such as counseled flats, flats for training in independent living and particular homes of people living in couples and alone. Regarding services, it is foreseen to improve fall detection and indoors location to provide ubiquitous services, to be put into the same prototyping level, integrated and tested.

## Figures and Tables

**Table 1.** Requirements for user profile, simplification of User Fit tool, template UA1, and UA2.

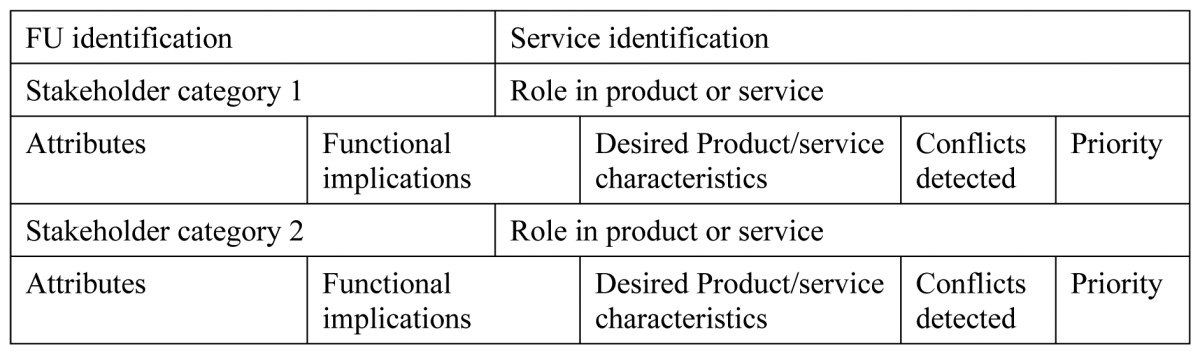

**Table 2.** Requirements for activity analysis, simplification of User Fit tool.

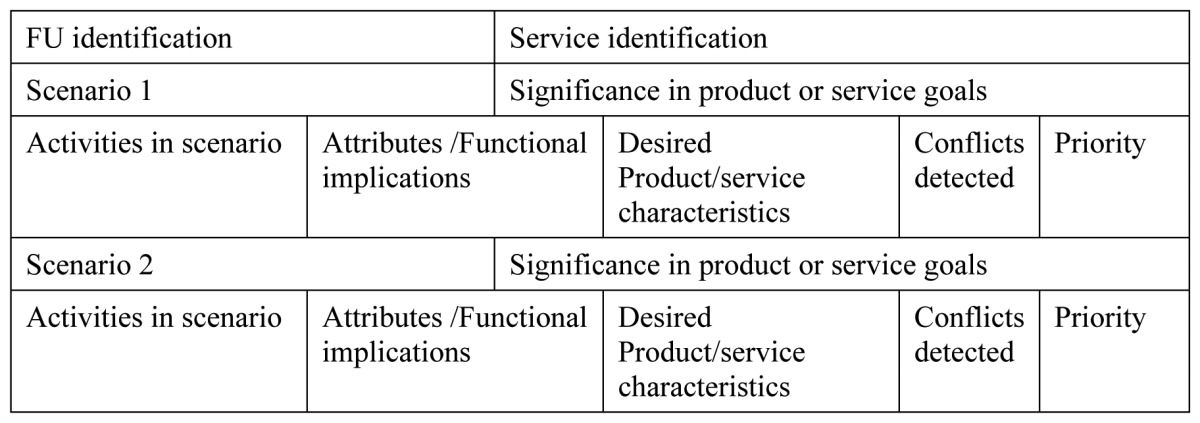

**Table 3.** Service description decomposing in framework layers the needed components.

**Alerts**	**Type**	**SP1 Modules used L2**	**SP1 Modules used L1**
Unusual presence detected (time of day, duration and place - presence sensor).	To be confirmed by SP1	E_UNPresence E-NoPresence (Toolbox)	S_location
When Vulnerable person is out of safe range	Core-research	E_OutArea (Toolbox)	S_Camera
When no movement is detected	Core-research	E_NoMove (Toolbox)	S_acceleration
Irregular Light Pattern (e.g. no light during all day and night)	Core-research	E_UNshutter (Toolbox)	S_light

**Table 4.** Human Machine Interface Service description decomposing in framework layers.

**HMI**	**Inputs**	**Outputs**	**SP1 Modules used L2**	**SP1 Modules used L1**
web page	✓	✓	O COM webpage I_COM_webpage	Webbrowser (mainstream)
via SMS		✓	O_COM_txt	Mobile-phone, HMI Assistive PDA
